# Clinical Application Research on Stroke Situational Intelligent Rehabilitation Training System Based on Wearable Devices: A Randomized Controlled Trial

**DOI:** 10.3390/healthcare13070708

**Published:** 2025-03-23

**Authors:** Ying Lu, Kangjia Ding, Yayuan Dai, Jie Yin, Jianjun Yao, Liquan Guo, Jiping Wang, Xiaojun Wang

**Affiliations:** 1 Suzhou Xiangcheng People’s Hospital, Suzhou 215131, China; 13915591820@163.com (Y.L.); monica2025dai@163.com (Y.D.); 15995887287@163.com (J.Y.); baiyi181@163.com (J.Y.); 2 Suzhou Institute of Biomedical Engineering Technology, Chinese Academy of Sciences, Suzhou 215010, China; dingkj@mail.ustc.edu.cn (K.D.); guolq@sibet.ac.cn (L.G.)

**Keywords:** stroke, rehabilitation training, motor function, ADL, wearable technology

## Abstract

**Background/Objectives:** With the advancement of intelligent sensing technology, rehabilitation systems based on wearable devices have a positive impact on the functional recovery and quality of life of stroke patients. This study aims to evaluate the application value of a contextualized intelligent rehabilitation training system for stroke survivors, which is based on wearable devices, in the rehabilitation of motor function impairments following stroke. **Methods:** A randomized controlled trial design was employed, in which 100 stroke patients were randomly divided into a control group (n = 50, receiving standard physical therapy rehabilitation interventions) and an experimental group (n = 50). The experimental group additionally underwent motor function rehabilitation interventions and intelligent assessments through a wearable device-based contextual intelligent rehabilitation training system, with sessions conducted twice daily for 30 min each, five days a week, over a duration of eight weeks. Both groups of patients underwent clinical scale evaluations and data collection before and after the treatment, with primary outcome measures including motor ability (Fugl–Meyer Assessment, FMA), activities of daily living (Modified Barthel Index, MBI), and participation in rehabilitation therapy. The intervention effects of both groups were compared after eight weeks of rehabilitation. **Results:** Prior to the intervention, there were no significant differences in Fugl–Meyer Assessment (FMA) and Modified Barthel Index (MBI) scores between the experimental group and the control group (*p* > 0.05). After eight weeks of rehabilitation, the experimental group demonstrated significantly superior performance in motor function (FMA scores) and activities of daily living (MBI scores) compared to the control group (*p* < 0.01). **Conclusions:** The intelligent rehabilitation system significantly enhances motor function and activities of daily living in stroke survivors. Compared to traditional rehabilitation methods, it improves patient adherence to rehabilitation training and overall outcomes.

## 1. Introduction

Stroke, commonly known as a cerebrovascular accident, is a neurological disorder characterized by vascular lesions in the brain. It stands as the second leading cause of death and the third leading cause of mortality and disability globally [1]. The pathophysiology of hemiplegia following stroke is complex, with long recovery periods for motor function impairment and a slow treatment process. Rehabilitation intervention after the acute phase of stroke is one of the key therapeutic approaches [2]. Upper limb dysfunction post-stroke is very common, persisting in approximately 70–85% of cases even 3 to 6 months later, which restricts patients’ independence and social participation, significantly reducing their quality of life and imposing substantial psychological and economic burdens on families and society [3]. Therefore, improving upper limb function to reduce disability and enhance activities of daily living (ADL) is a critical aspect of stroke rehabilitation [4,5].

Due to variations in the timing and methods of rehabilitation, as well as the lack of standardized home-based rehabilitation after discharge, there is a significant disparity in patient outcomes [6]. Thus, it is of great practical significance to manage hospital-based treatments and post-discharge home-based rehabilitation training effectively, continuously and systematically, to improve stroke-related neurological functions, enhance patients’ self-care abilities, and facilitate their maximum reintegration into society [7,8].

In hospital-based rehabilitation, healthcare professionals assess patients’ motor capabilities and provide scores based on assessment scales, which are conducted at regular critical intervals. However, these scales have limited predictive power [9], and the intermittent nature of such monitoring does not allow for accurate estimation of progress in motor activities, potentially missing opportunities for early intervention. Wearable devices, however, can provide rehabilitation training and continuous data collection for stroke patients, even in the absence of direct medical supervision, monitoring both their daily activities and adherence to rehabilitation plans [10]. The compact size of wearable technology ensures comfort for patients, avoiding discomfort or inconvenience, thereby encouraging active participation in rehabilitation exercises and activities.

Relevant studies have demonstrated that wearable sensors can achieve the functionality of assessing subjects’ activity types, intensity, duration, and quality. In a hospital setting [11], accelerometer sensors can be used to calculate an equivalent National Institutes of Health Stroke Scale (NIHSS) score, enabling the evaluation of motor recovery within the first 24 h post-stroke. Friedman et al. [12] utilized wearable sensors to determine the total amount of movement executed by the affected body parts. Arif et al. [13] monitored the physical activities of nine healthy subjects using three tri-axial accelerometers placed on the ankle, chest, and wrist. Lee et al. [14] employed a wrist-worn tri-axial accelerometer and gyroscope (Shimmer Ireland) to detect goal-directed (GD) upper limb movements during activities of daily living (ADLs), and further evaluated the quality of movement performance in subjects during home rehabilitation. Oubre et al. [15] estimated the severity of upper limb impairment using Fugl–Meyer Assessment (FMA) scores with two inertial measurement units (IMUs). Zhang et al. [16] developed a system for recognizing daily rehabilitation exercise motions. Yu et al. [17] proposed a wearable sensor network system for the quantitative monitoring and assessment of upper limb motor function.

Studies indicate that wearable devices offer significant assistance in the rehabilitation of motor function for stroke patients [18,19,20]. However, the benefits of context-aware rehabilitation training assisted by wearable devices, when compared to traditional treatments of the same frequency and duration, remain unclear. Some research [21,22] has reported on the mechanisms of wearable devices in recognizing movements and assessing rehabilitation in stroke patients, but there is still a lack of studies examining the impact of combining wearable devices with context-aware rehabilitation training on changes in motor function among stroke patients.

The COVID-19 pandemic has significantly accelerated the development of digital technologies in the healthcare sector. Notably, the use of tele-rehabilitation (TR) in stroke patient rehabilitation has been increasingly prevalent. Recent studies [23] have demonstrated that tele-rehabilitation after stroke is comparable to in-clinic rehabilitation in improving upper and lower limb motor functions and other stroke consequences. This growing trend highlights the importance of wearable devices in enabling remote rehabilitation services. In this context, our study aims to further explore the application of a stroke situational intelligent rehabilitation training system based on wearable devices and its potential advantages over traditional methods.

Therefore, this study aims to conduct a randomized controlled trial among post-stroke patients to compare the differences between situational intelligent rehabilitation training systems based on wearable devices and conventional clinical rehabilitation treatments, both provided at the same frequency and duration. The objective is to explore the effects and efficacy of integrating wearable devices with context-aware rehabilitation training on motor function in stroke patients. This research seeks to provide a reference for clinical rehabilitation practices, accelerating the recovery of hemiplegic stroke patients and facilitating their return to normal life as soon as possible.

## 2. Materials and Methods

A prospective, randomized controlled clinical trial was conducted. This study was approved by the Medical Ethics Committee of Xiangcheng People’s Hospital in Suzhou (Ethics Review Number: 2023-KY-01) and registered with the Chinese Clinical Trial Reg-istry (Registration Number: ChiCTR2300072642). Informed consent was obtained from all patients and their families. This study was carried out in accordance with the Declaration of Helsinki (Supplementary Materials).

### 2.1. Participants

From April 2023 to June 2024, we prospectively recruited 132 patients with post-stroke motor function impairment who were admitted to Xiangcheng People’s Hospital in Suzhou during the study period. The sample size was calculated based on the effect size from previous studies and the minimal clinically important difference [24,25,26]. Specifically, we used a standard sample size calculation formula with an effect size of 0.5 (based on the study by Guo et al. [25], where the effect size was determined by the ratio of the mean improvement score of the experimental group compared to the control group to their pooled standard deviation), a significance level of 0.05, and a statistical power of 80%. According to the estimated effect sizes from related stroke rehabilitation intervention studies, it was determined that a minimum of 30 participants per group would be required to detect significant differences in the primary outcome measure (Fugl–Meyer Assessment).

The inclusion and exclusion criteria were as follows:

Inclusion Criteria: (1) Diagnosis of stroke confirmed by CT or MRI, meeting the diagnostic criteria for stroke; (2) aged between 20 and 80 years; (3) patients who had a stroke more than 48 h but within six months prior to enrollment; (4) Brunnstrom stage II or higher; (5) able to follow the study protocol (Mini-Mental State Examination, MMSE score ≥24).

Exclusion Criteria: (1) significant cognitive deficits and unable to complete relevant as-assessments; (2) fractures, severe arthritis, amputations, or other significant limb impairments; (3) joint contractures, severe cardiac, hepatic, or renal insufficiency; (4) tumors, pregnancy, or coagulation disorders; (5) the presence of psychiatric or consciousness abnormalities and unable to cooperate with interventions.

**Figure 1:**
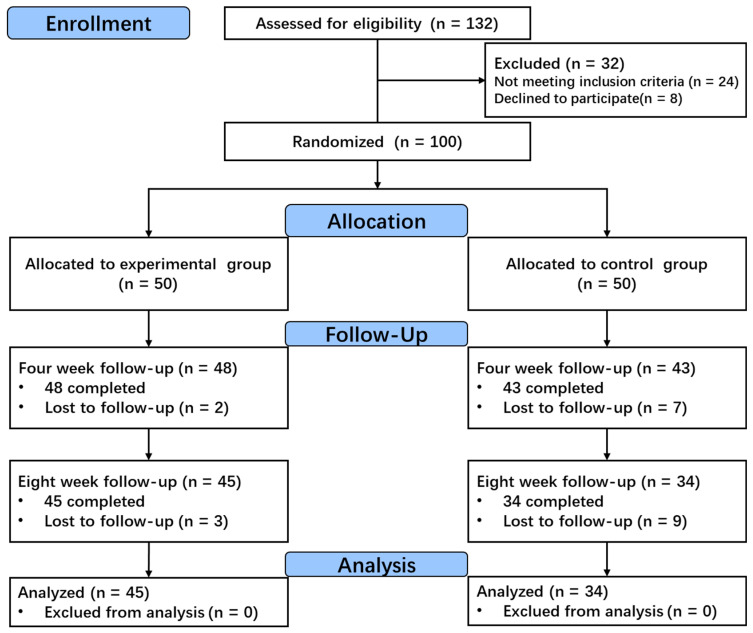
Flowchart of the selection process in this study.

Patients were randomly assigned to either the experimental group (n = 50) or the control group (n = 50) using a random number table. The experimental group received stroke situational intelligent rehabilitation training system combined with conventional rehabilitation therapy, while the control group received only standard treatment (Figure 1).

### 2.2. Study Design

**Figure 2:**
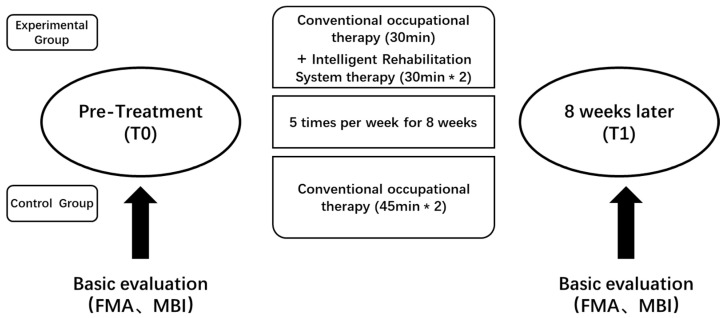
Study design flowchart.

All stroke patients admitted to the hospital and meeting the study criteria received motor function rehabilitation training, with all interventions conducted by experienced physical therapists. During this period, the experimental group (EG) received rehabilitation training using a context-aware intelligent rehabilitation system twice daily for 30 min each session, along with 30 min of conventional occupational therapy daily. This regimen was followed for 5 days a week over a period of 8 weeks. Participants in the control group (CG) received conventional rehabilitation therapy twice daily for 45 min each session, 5 days a week, for 8 weeks, following the same schedule as the experimental group. Motor function assessments were conducted at the beginning (T0) and end (T1) of the intervention, as detailed in Figure 2, which illustrates the study design flowchart.

#### 2.2.1. Stroke Situational Intelligent Rehabilitation Training System Based on Wearable Devices

The stroke situational intelligent rehabilitation training system based on wearable devices (Xi’an Libang Contmedu Medical Technology Co., Ltd., Xi’an, China) includes nine-axis inertial sensor modules for upper and lower limb rehabilitation, a rehabilitation glove for hand rehabilitation, and a Zigbee wireless receiver. Each IMU sensor module contains a nine-axis motion sensor, comprising a three-axis accelerometer, a three-axis gyroscope, and a three-axis magnetometer. During rehabilitation training, four IMU wearable sensors are attached to the upper arm, forearm, thigh, and calf. The rehabilitation glove is equipped with an IMU and five Flex sensors to monitor the movement of the wrist and individual fingers. The glove is designed for both left and right hands and comes in three sizes—large, medium, and small—to accommodate different patients.

**Figure 3:**
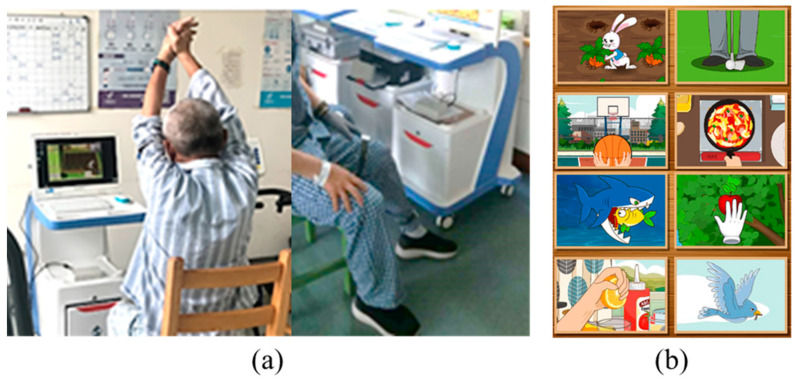
(**a**) Subjects are undergoing contextualized intelligent rehabilitation training. (**b**) Demonstration of the contextualized intelligent rehabilitation scenario.

The stroke situational intelligent rehabilitation training system based on wearable devices offers a rich set of features for context-aware intelligent rehabilitation. It helps stroke patients perform upper limb, hand, and lower limb motor function rehabilitation through contextualized games such as basketball shooting, making dumplings, and playing soccer. The system controls the contextualized rehabilitation games using limb movement characteristic data, enabling patients to engage in human–computer interactive training. It also provides visual and auditory feedback, effectively enhancing the motivation and enjoyment of stroke patients during rehabilitation training. See Figure 3 for details.

#### 2.2.2. Conventional Rehabilitation Training for Limb Motor Function

Physical therapists conduct rehabilitation training for the motor functions of the upper limbs, hands, and lower limbs of patients. Different training methods are selected according to the degree of limb motor dysfunction, including range of motion training for various joints, muscle strength training for the upper and lower limbs, balance and coordination training, and walking training, etc. In addition, training for activities of daily living such as eating and dressing is also carried out.

### 2.3. Outcome Measures

The primary outcome of this study was the improvement in limb motor function, as defined by the scores on the Simplified Fugl–Meyer Assessment (FMA) scale [27]. The FMA is composed of two dimensions: upper extremity (33 items) and lower extremity (17 items), totaling 50 items. Each item is scored from 0 to 2 points, with the total score calculated as the sum of the upper extremity score (ranging from 0 to 66 points) and the lower extremity score (ranging from 0 to 34 points). The overall score ranges from 0 to 100 points, where a higher score indicates milder motor dysfunction and better recovery [28,29].

The secondary outcomes included improvements in activities of daily living (ADL). ADL was assessed using the Modified Barthel Index (MBI) [30], which evaluates 10 activities related to daily living, such as eating, dressing, and transferring. Scoring is based on the need for assistance and the degree of help required, with the total score ranging from 0 to 100 points. A higher score signifies greater independence, less dependence, and better ADL performance [31].

The assessment results were obtained by a trained rehabilitation physician who was blinded to the patient group allocations and not involved in any other aspects of this study. During the assessment process, patient identity and group information were strictly confidential. The physician conducted objective measurements and recordings of the patients’ motor function using the Fugl–Meyer Assessment and their activities of daily living using the Modified Barthel Index, adhering strictly to the assessment criteria.

Participants showing “significant improvement” were defined as those whose FMA score increased by more than 20 points and MBI score increased by more than 15 points after the intervention. Participants with “almost no progress” were defined as those whose FMA score increased by less than 5 points and MBI score increased by less than 5 points.

### 2.4. Data Analysis

Data analysis was conducted using SPSS 26.0 software (Version 26.0; IBM Corp., Armonk, NY, USA) for statistical evaluation. Categorical data were represented by n and compared using the Chi-square (χ^2^) test. For continuous data that followed a normal distribution, means ± standard deviations (mean ± SD) were used to express the data. Paired sample *t*-tests were employed for within-group comparisons before and after treatment, while independent sample *t*-tests were used for between-group comparisons. In cases where data did not follow a normal distribution, non-parametric tests were applied, with results expressed as M (P25, P75). For these non-normally distributed data, paired Wilcoxon signed-rank tests were used for within-group comparisons, and the Mann–Whitney U test was applied for between-group comparisons. For ordinal data, the Mann–Whitney U test was also used for between-group comparisons, and the paired Wilcoxon signed-rank test was used for within-group comparisons. Statistical significance was set at *p* < 0.05 for all analyses.

To comprehensively evaluate the intervention’s effectiveness and address potential biases associated with participant dropout, this study also employed an Intention-to-Treat (ITT) analysis. All participants who were initially randomized were included in the ITT analysis. For participants who dropped out, missing data were handled using the Last Observation Carried Forward (LOCF) method. This approach helps ensure that the results more accurately reflect real-world conditions and minimizes selection bias.

In response to the reviewer’s feedback, we have incorporated additional data analysis methods, including Intention-to-Treat (ITT) analysis and Last Observation Carried Forward (LOCF) method. These analyses were not part of the original research design. The inclusion of these methods enhances the rigor of our research methodology. By adopting ITT analysis and LOCF approach, we aim to address potential biases and provide a more robust evaluation of the intervention’s effectiveness. This enhancement underscores our commitment to methodological thoroughness and the reliability of our findings.

## 3. Results

### 3.1. Baseline Characteristics of Participants

A total of 132 patients with stroke were screened at the Xiangcheng People’s Hospital in Suzhou City, among whom 100 met the eligibility criteria for this study and were assigned to either the experimental group (contextualized rehabilitation training for stroke) or the control group (conventional rehabilitation training). Twenty-one participants withdrew from treatment more than three times due to reasons unrelated to the type of therapy. In the experimental group, the main reasons for the dropouts were personal reasons (two participants) and health issues unrelated to the intervention (three participants). In the control group, the main reasons were lack of interest (eight participants), personal reasons (four participants), and health issues (four participants). Understanding these reasons can provide insights into the potential impact of dropouts on the study results.

We conducted a per-protocol analysis on data from 79 participants: 45 in the experimental group and 34 in the control group. Excluding participants who dropped out could potentially bias the results in favor of the experimental group and overestimate the effectiveness of the intervention. Therefore, an intention-to-treat (ITT) analysis was also performed, which included all participants initially randomized, regardless of whether they adhered to the intervention protocol. This approach allows for a more accurate assessment of the intervention’s actual effect, even in the presence of participant dropout, thereby enhancing the credibility and generalizability of the study conclusions.

By employing both per-protocol and ITT analyses, this study aims to provide a comprehensive evaluation of the intervention’s impact under real-world conditions, ensuring the reliability and robustness of the results interpretation.

For all participants who completed the entire trial, in the experimental group, there were 25 males and 20 females, with an average age of 61.45 ± 11.21 years; 18 cases had intracerebral hemorrhage, and 27 had cerebral infarction; the duration of illness was 1.40 ± 0.75 months. For the control group, there were 15 males and 19 females, with an average age of 63.33 ± 10.56 years; 13 cases had intracerebral hemorrhage, and 21 had cerebral infarction; the duration of illness was 1.45 ± 0.64 months. There were no statistically significant differences in the general information between the two groups of patients (*p* > 0.05), indicating comparability. Specific details are provided in Table 1. At baseline, both the experimental and control groups were homogeneous across all demographic and clinical features. Furthermore, all participants in the experimental group tolerated the training well, with no adverse events reported.

### 3.2. Comparison of FMA Scores Before and After Treatment Between the Two Groups

Prior to rehabilitation treatment, there was no statistically significant difference in FMA scores between the two groups (*p* > 0.05). Post-treatment, both the experimental and control groups exhibited a significant increase in FMA scores compared to pre-treatment levels (both *p* < 0.01). This finding suggests that both the intervention protocols applied to the experimental and control groups effectively improved motor function impairments in stroke patients. Eight weeks after the commencement of rehabilitation treatment, the FMA scores of patients in the experimental group were significantly higher than those in the control group (*p* < 0.01). This outcome indicates that the combination of a situational intelligent rehabilitation training system for stroke and traditional rehabilitation training is markedly more effective in enhancing motor function capabilities in stroke patients compared to conventional rehabilitation training alone. Detailed comparative results are presented in Table 2.

### 3.3. Comparison of Activities of Daily Living Before and After Treatment Between the Two Groups

Before treatment, there was no statistically significant difference in MBI scores between the two groups (*p* > 0.05). After treatment, both the experimental and control groups showed a significant improvement in MBI scores compared to their pre-treatment levels (both *p* < 0.01). This indicates that both the intervention protocols used in the experimental and control groups were effective in improving the ADL in stroke patients. Eight weeks after the initiation of rehabilitation treatment, the MBI scores of patients in the experimental group were significantly higher than those in the control group (*p* < 0.01). This result suggests that the combination of the situational intelligent rehabilitation training system for stroke and traditional rehabilitation training is significantly more effective in enhancing ADL in stroke patients compared to traditional rehabilitation training alone. Detailed comparative results are provided in Table 3.

### 3.4. Results of the Intention-to-Treat Analysis

Prior to the commencement of the trial, both the experimental and control groups consisted of 50 participants each, all of whom were enrolled in a randomized controlled trial. To minimize bias and accurately reflect the efficacy of the intervention, we performed an Intention-to-Treat (ITT) analysis, which included all participants who were randomly assigned, regardless of whether they completed the entire intervention or not. The purpose of conducting an ITT analysis was to reduce bias as much as possible and provide a more accurate assessment of the intervention’s effectiveness.

All 100 participants who were randomly assigned were included in the ITT analysis. For the 21 participants who dropped out during the trial (5 from the experimental group and 16 from the control group), the Last Observation Carried Forward (LOCF) method was utilized to handle missing data. The results of the ITT analysis are presented in Table 4. In terms of FMA scores, the difference between the experimental and control groups after treatment remained statistically significant (*p* < 0.01); similar results were observed with MBI scores, where the improvement in the experimental group post-treatment was significantly greater compared to that of the control group (*p* < 0.01).

## 4. Discussion

This study completed a randomized controlled clinical trial focusing on the rehabilitation of limb motor function in stroke patients. A total of 100 stroke subjects with varying degrees of limb motor dysfunction were included, aiming to investigate the feasibility of a wearable device-based situational intelligent rehabilitation training system for stroke, as well as its impact on limb motor dysfunction, and activities of daily living. The findings indicate that an 8-week intelligent rehabilitation training program for stroke is safe and feasible, with good completion rates among patients and no serious adverse events during the study period.

The current prospective randomized controlled trial results demonstrate that, compared to the control group receiving conventional rehabilitation training, stroke patients participating in the intelligent rehabilitation training program achieved significant improvements in limb motor function. This is evidenced by the better FMA score results in the experimental group compared to the control group when comparing the two groups after two months of post-stroke rehabilitation. Furthermore, the comparison of ADL outcomes between the two groups after 8 weeks of rehabilitation treatment showed that patients in the experimental group had significantly higher MBI scores than those in the control group, indicating that the use of the situational intelligent rehabilitation training system for stroke can lead to better performance in ADLs among stroke patients. These findings are consistent with previous research [32,33,34]. In addition to the per-protocol analysis, we conducted an Intention-to-Treat (ITT) analysis to include all randomized participants regardless of dropout. The ITT analysis showed that the experimental group still demonstrated significantly higher FMA and MBI scores compared to the control group, indicating that the intervention’s effectiveness is robust even when considering all randomized participants. This finding suggests that the situational intelligent rehabilitation training system for stroke is effective in improving motor function and activities of daily living in stroke patients, regardless of dropout rates.

Motor dysfunction resulting from stroke often requires prolonged rehabilitation to improve, yet most patients struggle to access long-term, effective rehabilitation treatments and personalized training programs. Residual functional impairments can significantly limit patients’ activities of daily living and reduce their quality of life [4]. A wearable sensor-based intelligent rehabilitation training system for strokes offer a novel and viable method for limb motor function rehabilitation. By providing scenario-based training tasks for the upper limbs, hands, and lower limbs, this system offers more targeted and individualized rehabilitation options for stroke patients, while also enhancing the interest and engagement in limb motor function rehabilitation [18,20,22]. Research findings indicate that a wearable device-based situational intelligent rehabilitation training system for stroke can effectively improve limb motor function, ADL, demonstrating significant superiority over single traditional clinical rehabilitation training methods.

Previous studies on the application of wearable devices in the rehabilitation of stroke patients have reached similar conclusions. Wei et al. [35] utilized wearable devices to generate vibrations to prompt patients to increase the use of their affected upper limbs, integrating functional activities of the upper limbs to promote the recovery of upper limb function in subacute stroke patients. Lee et al. [36] found that using a wearable hip-assist robot for gait training could effectively improve motor function and cardiopulmonary metabolic energy efficiency during walking in stroke patients. Lin et al. [37] demonstrated that the application of upper limb wearable devices in stroke rehabilitation could effectively increase the FMA scores and active range of motion in patients’ upper limbs. The wearable sensors of the intelligent rehabilitation training system for stroke can be adapted to upper limbs, hands, lower limbs, and the trunk, enabling comprehensive rehabilitation treatment and movement monitoring. Moreover, based on the data collected by the sensors, this system has developed a unique motor function evaluation system whose accuracy and effectiveness have been validated in prior research [25,38]. Additionally, integrating functional training with scenario-based virtual games can significantly enhance the interest and compliance of patients during the treatment process. Safety was also a key focus of this study. During training, the intelligent rehabilitation training system for stroke continuously monitors patients’ movements and provides real-time feedback and warnings; caregivers assist and protect patients during training, and therapists can monitor the training process face-to-face or remotely. Thanks to these measures, no adverse events, such as falls or pain, were reported throughout this study.

Our study indicates that the stroke situational intelligent rehabilitation training system based on wearable devices holds great promise for enabling remote rehabilitation at home. This is of particular significance as it can extend the reach of rehabilitation services beyond the clinic setting [23]. During this study, we observed that the portability of the wearable sensing devices allowed patients to potentially continue their rehabilitation training at home after discharge. However, several factors need to be considered for the successful implementation of home-based rehabilitation. These include ensuring patient compliance in the absence of direct supervision, providing adequate technical support to address any device-related issues, and establishing a reliable communication channel between patients and healthcare providers for continuous monitoring and guidance. Future research should focus on optimizing these aspects to fully realize the benefits of home-based rehabilitation in stroke recovery.

While appropriate statistical methods were utilized in our data analysis to mitigate the impact of outliers, it is important to acknowledge the presence of data points that may deviate from the mean. Such deviations can stem from individual differences among patients, including unique physiological conditions, subtle variations in stroke severity, or special events occurring during rehabilitation. For example, certain patients might exhibit markedly different recovery metrics due to comorbidities or unforeseen circumstances encountered during the rehabilitation period. Although these instances did not significantly alter the overall results of this study, future research should delve deeper into identifying and managing such data more effectively to enhance the accuracy and reliability of studies.

On the other hand, this study has limitations in the methodology used for data analysis. Although the per-protocol analysis demonstrated statistically significant improvements in motor function recovery (*p* < 0.01), we acknowledge that this approach may introduce selection bias by excluding participants with insufficient adherence. A post hoc intention-to-treat (ITT) analysis of all randomized subjects indicated a reduced improvement in limb motor function, suggesting that protocol adherence may enhance some of the treatment effects. This discrepancy highlights the need for future studies to predefine an ITT analysis framework supplemented by sensitivity analyses. It should be emphasized that the conclusions of this study remain valid within the per-protocol population. However, the generalizability of these findings to populations with lower adherence requires further validation.

We acknowledge that certain limitations are inherent in the post hoc analyses utilizing the intention-to-treat (ITT) approach and the last observation carried forward (LOCF) method. The ITT analysis may introduce bias by including participants who did not complete the intervention, while the LOCF approach risks overestimating the intervention’s efficacy. Despite these potential biases, our examination of group differences in dropout rates and baseline characteristics between the treatment and control groups suggests a relatively low risk of bias when employing the LOCF method for handling missing data. Future research should take these limitations into account and consider alternative methods for addressing missing data and conducting ITT analyses. Potential strategies include utilizing mixed-effects models for repeated measures (which can handle non-randomly missing data) and enhancing documentation on reasons for dropout (such as lack of efficacy or adverse reactions), thereby distinguishing mechanisms of missing data to further enhance the accuracy and reliability of study outcomes.

Initially, our research plan did not include the intention-to-treat (ITT) analysis and the use of the Last Observation Carried Forward (LOCF) method for handling missing data. These methods were added based on reviewers’ feedback to enhance the rigor of this study. We acknowledge that this is an area that could have been better designed in the original study and will be considered in future research.

Furthermore, this study did not conduct a specific analysis of participants who showed significant improvement or almost no progress, which are considered extreme cases. The reasons for such cases may include the severity of the initial stroke, compliance with rehabilitation, and individual differences. Participants who showed significant improvement may have benefited from a good physical foundation, a positive training attitude, and strong social support, while those who showed almost no progress may have been constrained by underlying diseases, poor training compliance, and other factors. Future studies should design specific plans to track and analyze these individuals to obtain more personalized practical outcomes, further refine rehabilitation treatment strategies, and provide more precise guidance for stroke patients with different rehabilitation progress. This study also has certain limitations. Firstly, due to the nature of the intervention, it was difficult to implement blinding for participants and treatment personnel, which inevitably introduces some bias. However, randomization was used to assign participants to groups, and assessors were blinded to the group assignments, efforts that were made to minimize the impact of bias on the study results. Secondly, the assessment was only conducted immediately after the intervention, without long-term follow-up observations.

## 5. Conclusions

Our study demonstrates that a wearable device-based situational intelligent rehabilitation training system for stroke may offer greater advantages in the rehabilitation and improvement of limb motor function in stroke patients compared to traditional conventional rehabilitation training. Specifically, wearable devices facilitate more convenient upper limb, hand, and lower limb motor function training for stroke patients. The application of smart sensors aids in recording patient movements and analyzing changes in data. Through these results, we can assert that stroke patients can benefit substantially from the situational intelligent rehabilitation training system in terms of limb motor function recovery. The ITT analysis further confirms the robustness of these findings, indicating that the intervention’s effectiveness is maintained even when considering all randomized participants.

The situational intelligent rehabilitation training system for stroke enables remote rehabilitation at home. The portability of wearable sensing devices allows patients to undergo effective and standardized rehabilitation training both during the initial phase of hospital treatment and after discharge in community settings and at home, which is of great significance for improving the effectiveness of rehabilitation treatment and deserves clinical promotion. In future research, we plan to apply this system to community and home-based rehabilitation for stroke patients to further explore its therapeutic effects in remote stroke rehabilitation.

## Figures and Tables

**Table 1:** Demographic and clinical characteristics of patients (per-protocol analysis).

	Control Group (n = 34)	Experimental Group (n = 45)	*p*-Value
Age, mean ± SD, y	63.33 ± 10.56 (38, 79)	61.45 ± 11.21 (35, 78)	0.45
Sex			
Male	15	25	0.42
Female	19	20	
Side of lesion			
Left	18	21	0.74
Right	16	24	
Type of stroke			
ICH	21	27	0.41
SAH	13	18	
Time of onset, mean ± SD, m	1.45 ± 0.64 (0.5, 6)	1.40 ± 0.75 (0.5, 6)	0.34

*p*-value: *t*-test (continuous variables); chi-square test or Fisher’s exact test (categorical variables); SD, standard deviation. ICH, intracerebral hemorrhage. SAH, subarachnoid hemorrhage. Data not presented as mean ± SD do not conform to a normal distribution.

**Table 2:** Comparison of FMA scores before and after treatment between the two groups.

	Control Group (n = 34)	Experimental Group (n = 45)	*p*-Value ^1^
FMA scores before treatment	36.34 ± 4.09 (26, 53)	37.26 ± 4.46 (25, 55)	0.344
FMA scores after treatment	52.57 ± 4.32 (40, 69)	64.83 ± 3.41 (46, 83)	0.001 *
***p*-Value ^2^**	0.001 *	0.001 *	

Values are presented as mean ± standard deviation (minimum and maximum); *p*-value ^1^: Wilcoxon signed-rank test, comparison before and after treatment by group; *p*-value ^2^: Mann–Whitney U test, between-group comparison of the pre-/post-treatment variation; * = statistically significant (*p* < 0.05).

**Table 3:** Comparison of ADL before and after treatment between the two groups.

	Control Group (n = 34)	Experimental Group (n = 45)	*p*-Value ^1^
MBI scores before treatment	54.53 ± 5.93 (40, 75)	55.27 ± 5.71 (40, 75)	0.58
MBI scores after treatment	65.47 ± 5.03 (50, 85)	78.73 ± 4.92 (60, 90)	0.001 *
***p*-Value ^2^**	0.001 *	0.001 *	

Values are presented as mean ± standard deviation (minimum and maximum); *p*-value ^1^: Wilcoxon signed-rank test, comparison before and after treatment by group; *p*-value ^2^: Mann–Whitney U test, between-group comparison of the pre-/post-treatment variation; * = statistically significant (*p* < 0.05).

**Table 4:** Comparison of intention-to-treat (ITT) analysis results.

	Control Group (n = 50)	Experimental Group (n = 50)	*p*-Value
FMA scores before treatment	36.04 ± 4.35 (24, 55)	37.55 ± 3.88 (25, 55)	0.412
FMA scores after treatment	49.37 ± 4.16 (33, 69)	64.45 ± 3.81 (39, 83)	0.001 *
MBI scores before treatment	54.46 ± 5.72 (37, 76)	55.12 ± 5.59 (38, 75)	0.562
MBI scores after treatment	63.82 ± 4.94 (43, 85)	77.92 ± 4.83 (50, 90)	0.001 *

Values are presented as mean ± standard deviation (minimum, maximum); *p*-Value: Wilcoxon signed-rank test, comparison before and after treatment by group; * = statistically significant (*p* < 0.05).

## Data Availability

The original contributions presented in this study are included in the article. Further inquiries can be directed at the corresponding author.

## Supplementary Materials

The following supporting information can be downloaded at: https://www.mdpi.com/article/10.3390/healthcare13070708/s1. CONSORT 2010 checklist of information to include when reporting a randomized trial.
